# Distinct 3D Structural Patterns of Lamin A/C Expression in Hodgkin and Reed-Sternberg Cells

**DOI:** 10.3390/cancers10090286

**Published:** 2018-08-24

**Authors:** Fabio Contu, Aline Rangel-Pozzo, Peter Trokajlo, Landon Wark, Ludger Klewes, Nathalie A. Johnson, Tina Petrogiannis-Haliotis, John G. Gartner, Yuval Garini, Roberta Vanni, Hans Knecht, Sabine Mai

**Affiliations:** 1 Cell Biology, Research Institute of Oncology and Hematology, University of Manitoba, CancerCare Manitoba, Winnipeg, MB R3E 0V9, Canada; fabio.contu89@gmail.com (F.C.); Aline.RangelPozzo@umanitoba.ca (A.R.-P.); umtrokaj@myumanitoba.ca (P.T.); lando.wark@gmail.com (L.W.); Ludger.Klewes@umanitoba.ca (L.K.); 2 Department of Biomedical Sciences, Unit of Biology and Genetics, University of Cagliari, 09042 Cagliari, Italy; vanni@unica.it; 3 Division of Hematology and Division of Pathology, Department of Medicine, Jewish General Hospital, McGill University, Montréal, QC H3G 2M1, Canada; nathalie.johnson@mcgill.ca (N.A.J.); tina.haliotis@mcgill.ca (T.P.-H.); hans.knecht@mcgill.ca (H.K.); 4 Department of Pathology, University of Manitoba, Winnipeg, MB R3E 0W2, Canada; John.Gartner@umanitoba.ca; 5 Physics Department & Institute of Nanotechnology, Bar Ilan University, Ramat Gan 5290002, Israel; Yuval.Garini@biu.ac.il

**Keywords:** Hodgkin’s Lymphoma, Reed-Sternberg cells, Lamin A/C patterns, 3D imaging, Lymphocytes, nuclear architecture

## Abstract

Classical Hodgkin’s lymphoma (cHL) is a B-Cell lymphoma comprised of mononuclear Hodgkin cells (H) and bi- to multi-nucleated Reed-Sternberg (RS) cells. Previous studies revealed that H and RS cells express lamin A/C, a component of the lamina of the nuclear matrix. Since no information was available about the three-dimensional (3D) expression patterns of lamin A/C in H and RS cells, we analyzed the 3D spatial organization of lamin in such cells, using 3D fluorescent microscopy. H and RS cells from cHL derived cell lines stained positive for lamin A/C, in contrast to peripheral blood lymphocytes (PBLs), in which the lamin A/C protein was not detected or weak, although its presence could be transiently increased with lymphocyte activation by lipopolysaccharide (LPS). Most importantly, in H and RS cells, the regular homogeneous and spherically shaped lamin A/C pattern, identified in activated lymphocytes, was absent. Instead, in H and RS cells, lamin staining showed internal lamin A/C structures, subdividing the nuclei into two or more smaller compartments. Analysis of pre-treatment cHL patients’ samples replicated the lamin patterns identified in cHL cell lines. We conclude that the investigation of lamin A/C protein could be a useful tool for understanding nuclear remodeling in cHL.

## 1. Introduction

Classical Hodgkin’s lymphoma (cHL) is characterized by two tumor cell populations: mononuclear Hodgkin cells (H) and bi- to multi-nucleated Reed-Sternberg cells (RS), which are the diagnostic cells of this lymphoma. H and RS cells represent only 1–5% of the total lymph node cells [1]. H cells originate from crippled, pre-apoptotic germinal center B cells [2] that have been rescued from apoptosis by cellular transformation events [3,4]. RS cells originate from H cells through a process of multinucleation due to incomplete cytokinesis [5]. Transition from H to RS cell has also been correlated with an aberrant number of mitotic spindles, increased numbers of centrosomes, altered 3D telomere organization and telomere loss [6]. Advanced telomere loss and aggregation result in RS cells presenting telomere-poor ‘ghost’ nuclei, which interfere with endomitosis [7]. Super resolution microscopy (3D-structured illumination microscopy, 3D-SIM) investigation showed that the nuclear DNA structure is significantly changed in H and RS cells when compared to normal primary lymphocytes, with a significant increase in the amount of DNA-free or DNA-poor nuclear spaces defined by the absence of 4’,6-diamidino-2-phenylindole (DAPI)-staining [8,9]. The number of DAPI-free spaces is increased in cancer cells during the transition from H to RS cells [8].

Lamins are type V intermediate filament (IF) proteins, which are the principal component of the lamina of the nuclear matrix. Two types of lamin have been described in human cells: B-type lamins, encoded by the *LMNB1* gene for lamin B1 [10] and the *LMNB2* gene for lamin B2 [11], and A-type lamins, encoded by the *LMNA* gene, the alternative splicing of which produces lamin A and lamin C [12]. Lamin B1 and lamin B2 are constitutively expressed and necessary for cell survival [13]. Lamin A/C expression differs from cell to cell and is usually limited to differentiated cells and not found in proliferating cells [14]. Lamin proteins are involved in a myriad of nuclear processes, including DNA replication, RNA transcription, cell differentiation and mitosis [15]. In particular, lamin A/C plays a crucial role in the regulation of mitotic spindle assembly and positioning [16].

Resting human and mouse T lymphocytes express lamin A/C, and its presence is transiently and considerably increased upon T cell activation [17]. Lamin A/C expression has been found to be down-regulated in different cancer types, like small cell lung cancers [18], colon cancers [19] and nodal diffuse large B-cell lymphoma [20]. On the other hand, squamous cell carcinoma and basal cell carcinoma are characterized by an up-regulation of lamin A/C [21]. Investigation of lamin A/C expression in neuroblastoma [22] and in prostate cancer [23] has been proven to be a reliable biomarker of cancer aggressiveness.

The first investigation of lamin proteins in reactive lymph nodes and cHL samples showed that lamin A/C was not expressed in CD20+ non-neoplastic B lymphocytes, but that it was expressed by a large population of CD30+ cells, in nine patients with nodular sclerosis Hodgkin’s lymphoma [24].

To our knowledge, no data have been reported on 3D lamin A/C protein expression patterns in the H and RS cells of cHL patients and their relation to the process of multinucleation, namely the transition of cellular architecture from H to RS cells. Also, no data was reported on B lymphocyte lamin A/C expression following activation.

In this study, we investigated the three-dimensional (3D) spatial distribution of lamin A/C in three different cHL derived cell lines, in resting and stimulated purified peripheral blood lymphocytes (PBLs); and in 12 primary paraffin-embedded pre-treatment lymph node samples from patients diagnosed with cHL. Our findings reveal, for the first time, the presence of an aberrant lamin A/C structure in H and RS cells, which is distinct from that seen in normal lymphocytes.

## 2. Results

### 2.1. Lamin A/C and Lamin B1 in Hodgkin Lymphoma Derived Cell Lines and PBLs

**Figure 1:**
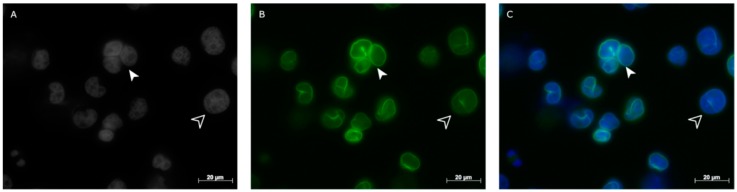
Example of lamin A/C protein staining in cells from Hodgkin’s lymphoma (HDLM-2). (**A**) Two-dimensional (2D) image of nuclei stained with 4′,6-diamidino-2-phenylindole (DAPI)-(gray scale); (**B**) 2D image of anti-lamin A/C antibody immunostaining (green); (**C**) 2D merged image showing both mono-nuclear H (empty arrowhead) and bi- to multi-nuclear RS cells (solid arrowhead) expressing lamin A/C.

To assess lamin A/C positivity in H and RS cells we performed immunostaining for lamin A/C and lamin B1 in three cHL-derived cell lines and normal lymphocytes. Immunohistochemical analysis revealed that H and RS cells from all the HL-derived cell lines stained for both lamin A/C (Figure 1A–C) and lamin B1 (Figure S1A–E).

**Figure 2:**
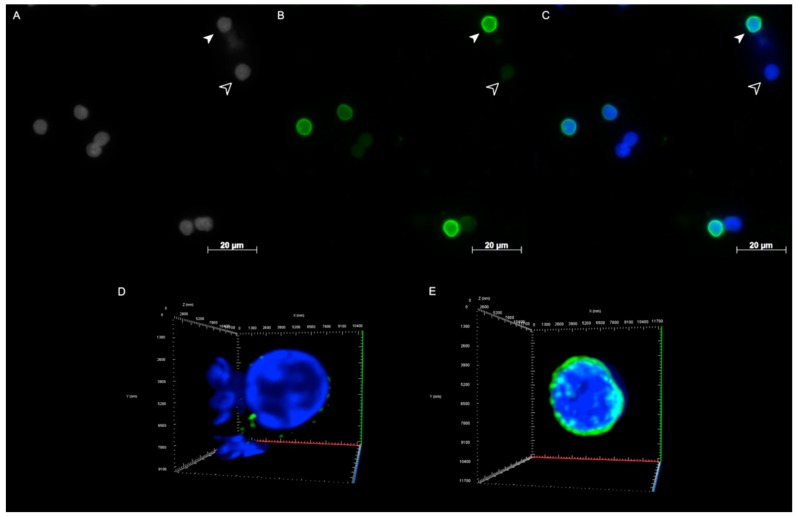
Lamin A/C immunostaining of resting and LPS-activated lymphocytes from peripheral blood (PB) of a healthy donor. (**A**) 2D image of nuclei stained with DAPI (gray scale); (**B**) 2D image of anti-lamin A/C antibody immunostaining (green); (**C**) 2D merged image showing activated lymphocytes with a higher fluorescence intensity lamin A/C signal (solid arrowhead) compared to resting cells (empty arrowhead). Complete 3D reconstitution of a resting (**D**) and an activated (**E**) lymphocyte.

As healthy controls, we used both lipopolysaccharide (LPS)-activated and resting normal B lymphocytes (Figure 2A–C). Resting PBLs showed weak to no positivity for lamin A/C expression (Figure 2D), while they were positive for lamin B1 (Figure S2A–D). However, lamin A/C expression increased after B cell activation with LPS (Figure 2E).

### 2.2. Lamin A/C and Lamin B1 3D Spatial Distribution Patterns in Hodgkin Lymphoma Derived Cell Lines and PBLs

The lamin A/C 3D spatial distribution in resting PBLs was characterized by points of protein accumulation (Figure 2D). The lamin A/C pattern in activated lymphocytes appears as a regular sphere surrounding the nucleus (Figure 2E). The regular pattern is replaced in H and RS cells by a more irregular one, characterized by the presence of internal lamin A/C structures, dividing the 3D structure into multiple compartments not seen in the normal lymphocytes.

We identified five different types of lamin A/C patterns for H cells (0, A, B, C and D), and four for the RS cells (bi-nuclear, tri-nuclear, tetra-nuclear and multi-nuclear) (Table 1; Table S1). Quantitative analysis of the identified patterns for both H and RS cells was performed investigating the ratio of the external total lamin A/C intensity (I_e_) on the internal total lamin A/C intensity (I_i_) (Table 2).

**Figure 3:**
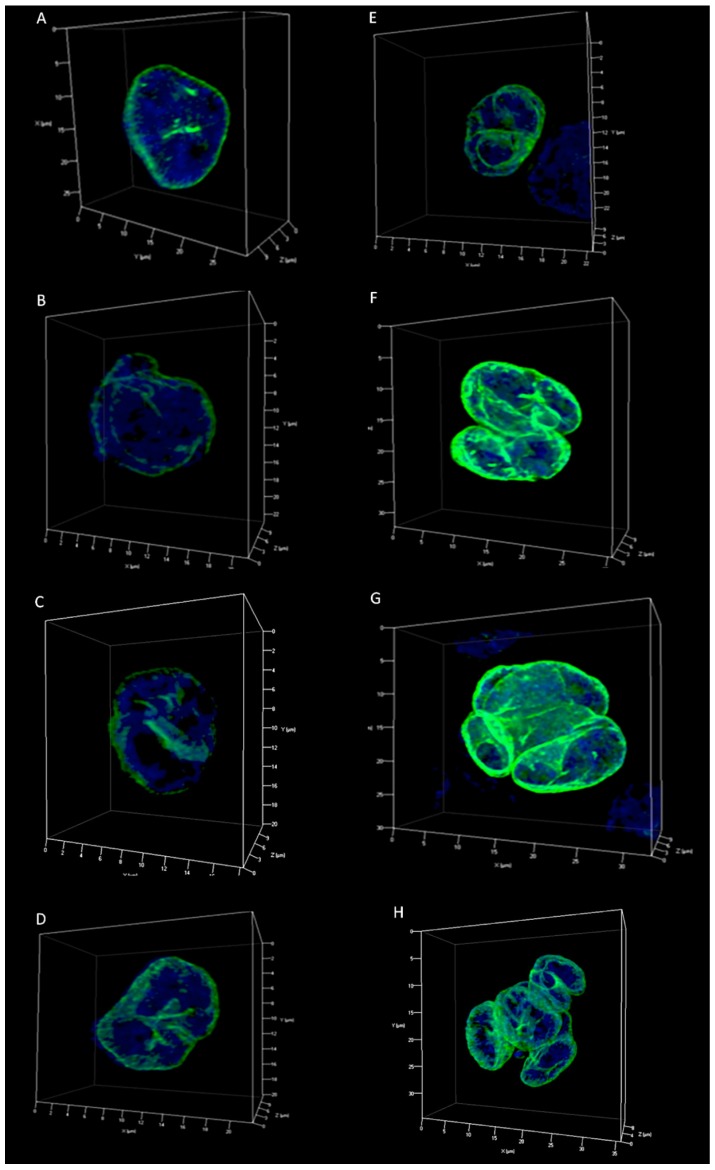
Lamin A/C patterns in 3D reconstructions of H and RS nuclei from HDLM-2. (**A**–**E**) H cells patterns according to how the internal lamin structures divide the 3D structure of lamin A/C: (**A**) pattern 0, characterized by a 3D pattern similar to the regular pattern of the LPS-activated lymphocytes, showing, however, localized accumulation of the lamin A/C; (**B**) pattern A, characterized by the irregular lamin A/C 3D distribution and the presence of invaginations due to short internal lamin structures; (**C**) pattern B, characterized by a single long internal lamin A/C 3D structure, which divides the nucleus into 2 different compartments; (**D)** pattern C, characterized by 3D multiple internal lamin structures which divide the nucleus into 3 different compartments; (**E**) pattern D, characterized by 3D division of the nucleus into 4 different compartments. (**F**–**H**) RS cells patterns according to the number of nuclei: (**F**), bi-nuclear RS cell; (**G**) tetra-nuclear RS cell; (**H**) multi-nuclear RS cell.

The lamin A/C patterns were observed in all three HL-derived cell lines analyzed in this study. H cells were divided into groups based on how the lamin A/C 3D internal structures divided the nucleus (Figure 3A–E, Videos S1–S5). Pattern 0 has similar architecture to that found in LPS-activated normal B cells (Figure 3A). Pattern A is characterized by the presence of small internal structures and by the irregular 3D profile of lamin A/C (Figure 3B). In Pattern B, C, and D, the aberrant lamin A/C staining divides the 3D structure of mono-nuclear H cells into two, three and four different compartments, defined by internal lamin structures (Figure 3C–E). Mononuclear cells expressing pattern B constituted a majority (42.16%), followed by patterns A (29.15%), 0 (15.48%), C (11.63%) and D (1.59%). The pattern classification for H cells described increasing complexity of the internal lamin A/C structure and the quantitative analysis reflected this change as the I_e_/I_i_ ratio increased.

RS cells were divided into different groups according to the numbers of total nuclei, all covered by a spherical lamin surface. However, lamin A/C showed a more irregular pattern when compared to the mononuclear H cells. Anomalous lamin A/C features observed in H cells were also present (Figure 3F–H, Videos S6–S8). Bi-nuclear RS cells were found to be the most frequent (57.66%) in the RS cell population analyzed, followed by tri- (22.27%), tetra- (10.29%) and multi-nuclear (9.79%) RS cells. In RS cells as the pattern classification complexity of the lamin A/C structures increases from bi- to multi-nuclear, the quantitative analysis reflects this change with an increasing I_e_/I_i_ ratio.

Investigation of lamin B1 3D spatial distribution revealed the presence of the same types of patterns identified for lamin A/C in all the three HL-derived cell lines (Figure S1D,E), which were, however, observed with different frequencies (Table S2). The differences were statistically significant (respectively, H: *p* = 0.0002; RS: *p* = 0.006).

### 2.3. Hodgkin’s Lymphoma Patient Samples and Reactive Tonsils Samples

**Figure 4:**
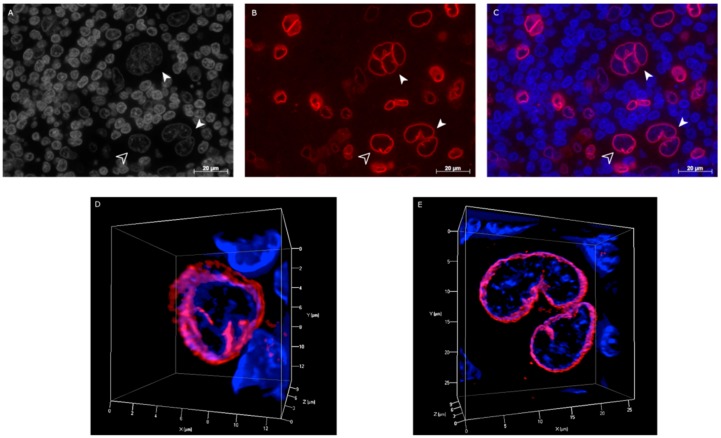
Example of lamin A/C expression in primary Hodgkin’s lymphoma paraffin-embedded pre-treatment lymph node tissues from patients diagnosed with cHL. (**A**) 2D image of nuclei stained with DAPI (gray scale); (**B**) 2D image of anti-lamin A/C antibody immunostaining (red); (**C**) 2D merged image showing H (empty arrowhead) and RS (solid arrowhead) cells positively stained for lamin A/C. 3D reconstruction of patient-derived mono-nuclear Hodgkin (H) (**D**), and bi-nuclear (RS) cells (**E**), presenting the same patterns of irregular lamin A/C 3D structure characterized by internal lamin structures and points of protein accumulation, similar to those seen in the HL derived cell lines.

Twelve primary diagnostic classical Hodgkin’s lymphoma pre-treatment lymph node biopsy tissue sections were immunostained for lamin A/C. CD30 positivity was used to recognize H and RS (Figure S3A–C). Immunohistochemical analysis revealed that the majority of the non-neoplastic lymphocytes in the lymph node were lamin A/C negative, while all H and RS cells stained positively for lamin A/C. However, the lamin A/C fluorescent signal was not consistent throughout the patients’ samples. The levels of the lamin A/C fluorescent signal was assessed as follows: (i) −/− (poor), patient samples with weak fluorescent signals, in which it was impossible to evaluate lamin A/C 3D spatial distribution of H and RS cells; (ii) −/+ (average), patient samples with strong lamin A/C staining and clear 3D spatial distribution in a limited number of H and RS cells; (iii) +++ (high), patient samples with strong lamin A/C staining and clear 3D spatial distribution in most of the H and RS cells (Table 3). H and RS cells from patient samples with high lamin A/C fluorescent signal intensity presented the irregular 3D lamin A/C staining pattern characterized by internal lamin structures and points of protein accumulation (Figure 4). The different lamin A/C patterns observed in the H and RS cells of the Hodgkin cell lines were also identified in the H and RS cells of patient samples (Figure 4D,E). The majority of H cells expressed pattern 0 (45.71%) or pattern A (34.29%). Patterns B (14.29%) and C (5.71%) were rarely observed.

To assess lamin A/C spatial distribution in the germinal center (GC) lymphocytes, precursors of the H cells, five reactive tonsils biopsy tissue sections were immunostained for lamin A/C. Immunohistochemical analysis of the GC revealed weak-to-no positivity for lamin A/C (Figure S4A–C). Analysis of the centrocytes within the dark zone (DZ) of the GC confirmed the absence of lamin A/C staining (Figure S4D–G).

### 2.4. Co-Localization of Lamin A/C and Telomeres in H and RS Cells

To investigate the interaction of lamin A/C and telomeres, we combined the immuno-staining for lamin A/C with telomere quantitative fluorescent in situ hybridization (Q-FISH). The deconvolved 3D cell reconstructions of 30 H and 30 RS cells from three independent experiments were loaded on ImageJ (NIH, Bethesda, MD, USA) and analyzed through the TANGO (Sorbonne University, Paris, France) plug-in [25,26]. The overlapping percentage of the Cy3 signals of the telomeres and the fluorescein isothiocyanate (FITC) signal of the internal lamin A/C structures was examined by putting the generated data through a binary logic gate: a value of 0 was assigned by the software when no overlap was detected between a telomere signal and lamin A/C; a value of 1 was assigned when overlap was detected. The analysis revealed that only a minority of the telomeres co-localized with lamin A/C in the H cells (mean 33.06%; S.D. 0.08) and in the RS cells (mean 38.11%; S.D. 0.07) respectively.

### 2.5. Silencing of Lamin A/C mRNA and 3D Telomere Structure Analysis

**Figure 5:**
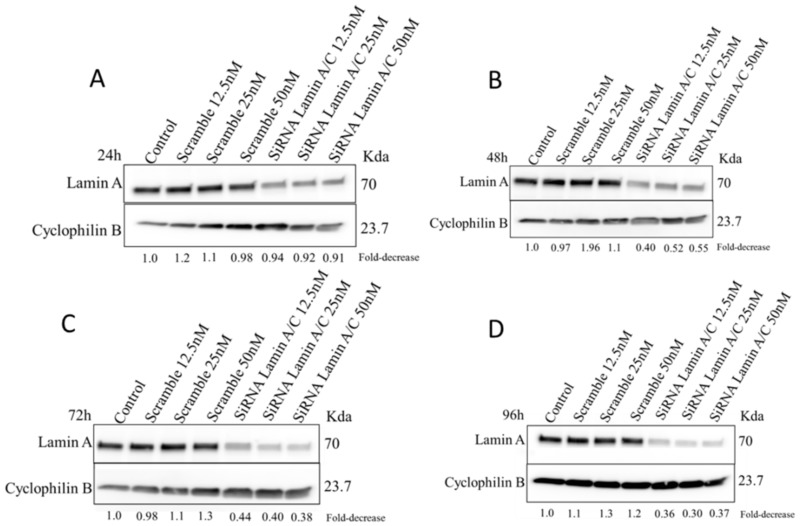
siRNA silencing of lamin A/C in HDLM-2. Lamin A/C expression was monitored by Western Blot after siRNA lamin A/C transfection in different concentration (12.5 nM, 25 nM and 50 nM) for 24 h (**A**), 48 h (**B**), 72 h (**C**) and 96 h (**D**). siRNA Scramble was used as a negative control and Cyclophilin B was used as a loading control. The fold-decrease for the siRNA is relative to the negative control (Scramble for each concentration) and the Scramble is relative to control without transfection (Control). These experiments were performed in triplicate using cells from different passages.

**Figure 6:**
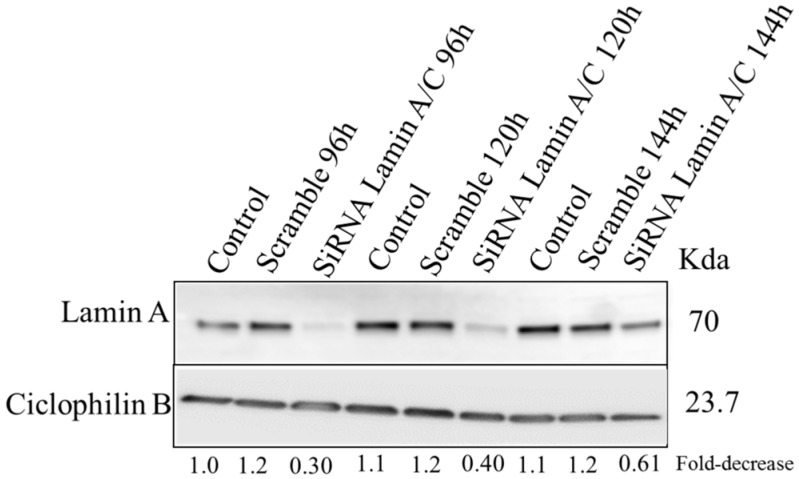
Investigation of siRNA silencing of lamin A/C in HDLM-2 for additional time points. Lamin A/C expression was monitored by Western Blot after siRNA lamin A/C transfection for 96 h, 120 h and 144 h (50 nM). siRNA Scramble was used as a negative control and Cyclophilin B was used as a loading control. The fold-decrease for the siRNA is relative to the negative control (Scramble for each concentration) and the Scramble is relative to control without transfection (Control). These experiments were performed in triplicate using cells in different passages.

In order to explore the effect of lamin A/C silencing in the HDLM-2 cells in terms of multinucleation process and genomic instability, we transfected the HDLM-2 cells with siRNA lamin A/C and non-targeting siRNA (Scramble) in three different concentrations for 24 h, 48 h and 72 h. Lamin A/C silencing was evaluated by Western Blot (WB) (Figure 5) which revealed a decrease in lamin A/C expression after 24 h with an even greater decrease after 96 h compared to control cells (non-targeting siRNA named as Scramble and cells without transfection named as control). After 96 h, the expression of lamin A/C was down-regulated by about 70% (Figure 5), and the number of RS cells decreased from 48.37 to 25% in the HDLM2 siRNA lamin A/C when compared to the Scramble. Subsequent time points of 120 h and 144 h were investigated to understand the complete kinetics of siRNA-mediated lamin A/C silencing and its effects in the 3D nuclear organization of telomeres (Figure 6). After 96 h, the degradation of complementary lamin A/C mRNA molecules started to decrease as shown in Figure 5. To analyze the effects of siRNA on the 3D nuclear organization of telomeres we combined the immuno-staining for lamin A/C with Q-FISH.

Nuclei of 30 H and 30 RS cells transfected with siRNA lamin A/C were used for the telomere analysis. Scramble H and RS cells were used as controls. siRNA treated H cells, after 96 h, were characterized by a decrease in the average telomere signal intensity (*p* = 0.0119), and by changes in the overall telomere spatial organization, as demonstrated by an increased a/c ratio, when compared to the control (*p* = 0.0079). The increased a/c ratio was also observed after 120 h (*p* = 0.0038) and 144 h (*p* = 0.0015). siRNA treated RS cells, after 96 h, were characterized by an increase in the total number of telomeres (*p* = 0.0013), an increase in number of telomere aggregates (*p* = 0.0073), an increase in nuclear volume (*p* < 0.0001), and a decrease in average telomere signal intensity (*p* = 0.0006) when compared to the Scramble control. The nuclear volume was still increased after 120 h (*p* < 0.0001) and 144 h (*p* = 0.0057).

Moreover, the comparison among the 96 h, 120 h and 144 h time points revealed other telomere related abnormalities. In siRNA treated H cells, while both the total number of signals (*p* = 0.0308) and the total number of aggregates (*p* = 0.0473) increase from 96 h to 144 h, the average telomere intensity (*p* = 0.0081), the total telomere signal intensity (*p* = 0.0311), the a/c ratio (*p* < 0.0001) and the nuclear volume (*p* < 0.0001) decrease from 96 h to 144 h. Total number of telomere signals (*p* = 0.0280) and the total number of telomere aggregates (*p* = 0.0208) are found to be increased also in siRNA treated RS cells after 144 h when compared to 96 h, and the average telomere intensity (*p* = 0.0002), the total telomere signal intensity (*p* = 0.0318) and the nuclear volume (*p* = 0.0027) decrease as observed in the H cells. Prolonged downregulation of lamin A/C induced genomic instability in both H and RS cells, suggesting a prominent role of lamin A/C in the maintenance of telomere 3D spatial organization.

### 2.6. DNA Structure and Stucture of DNA-Poor Spaces in siRNA Treated HL Cell Line

**Figure 7:**
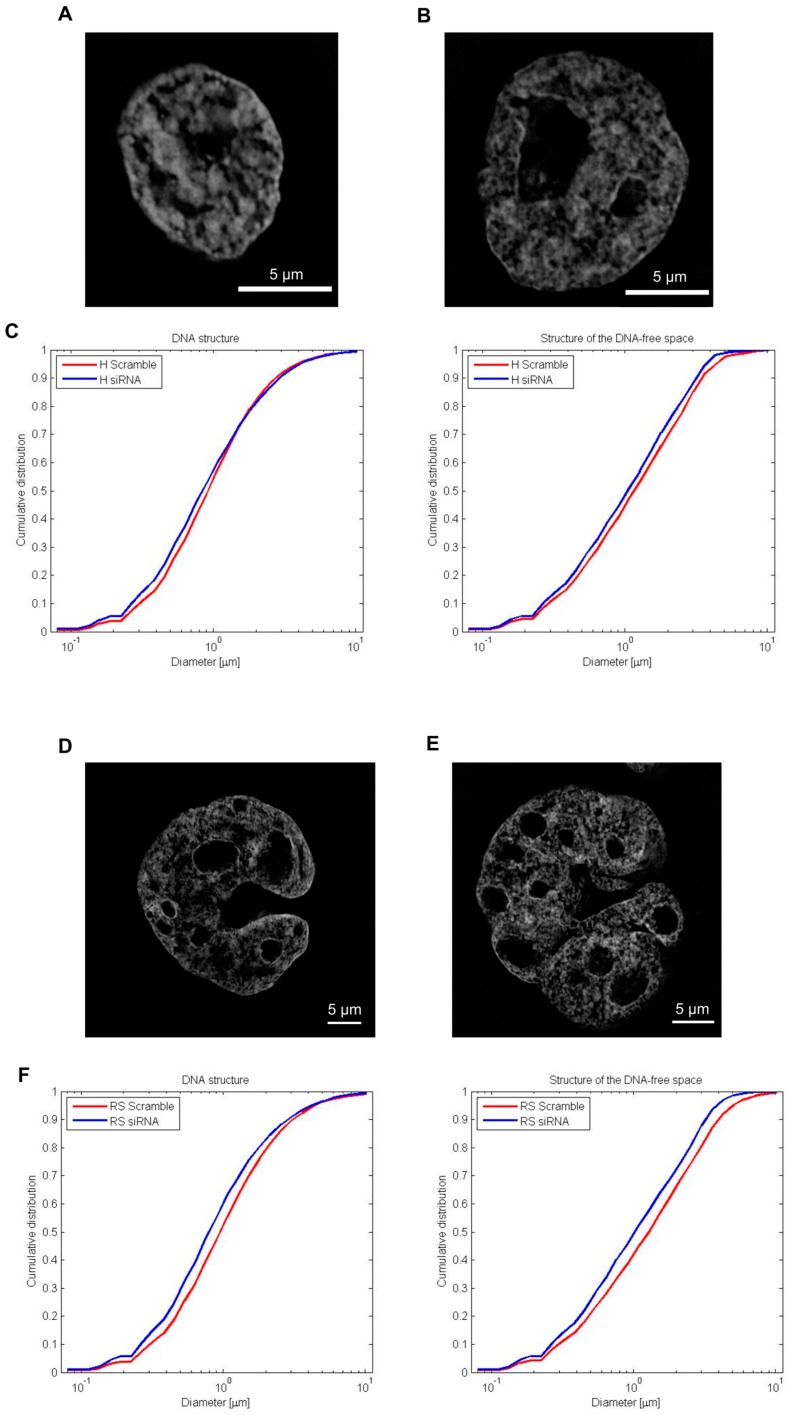
Granulometry analysis of siRNA lamin A/C cells and Scramble cells: (**A**) SIM image of Scramble H cell; (**B**) SIM image of siRNA H cell; (**C**) Measurements of the size distribution of DNA structure and structure of the DNA-free space in SIM images of Hoechst (33258) stained nuclei of 30 H cells from three independent experiments; (**D**) SIM image of Scramble RS cell; (**E**) SIM image of siRNA RS cell; (**F**) Measurements of the size distribution of DNA structure (H: *p* = 0.26; RS: *p* < 0.001) and structure of the DNA-free space (H: *p* = 0.37; RS: *p* < 0.001) in SIM images of Hoechst (33258) stained nuclei of 30 RS cells from three independent experiments. Two-sided, two-sample Kolmogorov–Smirnov test was used to determine statistical significance.

Structured illumination microscopy (SIM) of 30 H and 30 RS cells from three independent siRNA lamin A/C samples (50 ng, 96 h) (Figure 7B,E respectively) and Scramble controls (Figure 7A,D respectively) were used to compare the 3D structural organization of the nuclear DNA of the two experimental arms. We used a granulometry-based measurement technique to quantify both the DNA structure and the structure of DNA-free space inside interphase nuclei [8]. The cumulative distributions of structure sizes (Figure 7C,F) have been plotted. Two-sided, two-sample Kolmogorov–Smirnov test showed that although the size distribution of both the DNA structure (*p* = 0.26) and DNA-free space (*p* = 0.37) was not significantly different between siRNA transfected H cells and Scramble H cells, the comparison between siRNA transfected RS cells and Scramble RS cells showed a difference. Both the DNA structure (*p* < 0.001) and structure of the DNA-free space (*p* < 0.001) contained finer structure for siRNA lamin A/C cells, suggesting a prominent role of lamin A/C in the nuclear architecture maintenance.

## 3. Discussion

Our study is the first one to describe different lamin A/C 3D nuclear organizational patterns leading to progressively complex nuclear matrix compartmentalization by laminar structures in H and RS cells in cHL-derived cell lines and in patients’ diagnostic pre-treatment lymph nodes that are distinct from what is seen in normal lymphocytes, and in non-neoplastic (inflammatory) lymphocytes in the same lymph nodes.

H and RS cells from classical Hodgkin’s lymphoma-derived cell lines showed high lamin A/C expression. In contrast, isolated resting PBLs were lamin A/C negative or presented weak lamin A/C staining. Immunohistochemical analysis confirmed that LPS-activated B cells stained positively for lamin A/C and were used as a control for H and RS cells from HL-derived cell lines.

It has been demonstrated that dysregulation of lamin A/C can affect DNA transcription, replication and repair, inducing genomic instability which leads to cancer progression [27,28,29]. Lamin A/C, in fact, plays a role in mitosis by regulating mitotic spindle assembly and positioning [16]. Since RS cells are characterized by an increased number of mitotic spindles, and incomplete spindles are regularly detected [6], lamin A/C could be involved in the multinucleation process. In order to verify the role of lamin A/C in this process, we performed silencing experiments of the lamin A/C mRNA through siRNA transfection. The number of RS cells after 96 h was found to be decreased. It has been demonstrated that telomere loss and aggregation in RS cells interferes with the endomitotic process [7]. Telomere analysis through the different time points showed that siRNA treated H and RS cells were characterized by an increased genomic instability, with an increase in the number of short telomeres and telomere aggregates, revealing that lamin A/C is crucial for the multinucleation process. In addition, silencing of lamin A/C led to a progressive disruption of nuclear DNA organization in siRNA lamin A/C treated cells when compared to the control, suggesting that lamin A/C plays an important role in determining the nuclear structure.

Our findings indicated that H and RS cells had an irregular lamin 3D distribution pattern compared to the 3D structure of lamin A/C in LPS-activated lymphocytes, which displayed a continuous spherical protein matrix surrounding the nucleus. Specifically, H and RS cells were characterized by the presence of lamin A/C invaginations, points of protein accumulation, and internal lamin structures that subdivide the nuclei into multiple compartments. According to the organization of the internal structures and number of resulting sub-compartments, we defined five different groups for the H cells (0-A-B-C-D), and four different groups for the Reed-Sternberg cells (bi-nuclear, tri-nuclear, tetra-nuclear and multi-nuclear). Pattern 0 of H cells is morphologically similar to the regular one characteristic of LPS-activated lymphocytes, except for localized accumulation of lamin A/C. Pattern A is characterized by an irregular lamin A/C 3D spatial distribution and the presence of invaginations and short internal lamin structures. Pattern B presents one long lamin internal structure, which divides the H cell nuclei into two distinct compartments. We supposed that this lamin A/C compartmentalization could anticipate the transition of a mono-nuclear H to a bi-nuclear RS cell, making lamin A/C a useful tool to predict the future behavior of the cell. Pattern C is characterized by the presence of multiple internal lamin structures which divide the cell into three different portions, either foreshadowing a transition from H to tri-nuclear RS cell or indicating that this is an end-stage H cell unable to perform further nuclear division. Pattern D shows a division into four different regions and could either anticipate a transition from a mono-nuclear H to tetra-nuclear RS cell, or again indicate an end-stage H-cell no longer capable of dividing.

To investigate the above hypothesis, we divided the H and RS cells from three independent experiments (90 cells total for each cell type) into different groups according to which specific pattern they showed. The obtained percentages are consistent with the hypothesis that a significant proportion of mono-nuclear H-cells is still capable of progression to RS-cells [30]. However, considering that likely not every mono-nuclear H cell will complete the transition to a RS cell [6], lamin A/C may be a useful tool in predicting the future behavior of an H cell and, therefore, of the neoplastic population of any given patient on the whole.

The analysis of the HL patient samples confirmed the presence of the patterns we observed in the Hodgkin cell lines, with most of the H cells expressing patterns similar to the one of the activated lymphocytes. More complex ones, like pattern B or pattern C, were observed with lower frequency. Though in histological slide preparations the thickness of the cut is 5 µm, and, therefore, never includes the whole nuclear volume of H- and RS-cells, our collective data suggest that the observed differences in lamin A/C spatial organization is likely to be linked to an alternative, aberrant remodeling of the lamin A/C protein in H and RS cells, as compared to normal lymphocytes. However, a larger cohort of patients will be required to confirm the distribution of lamin A/C patterns among the H and RS cell population in the lymph node.

It is known that lamin A/C plays a crucial role in maintenance of genome organization, since it is responsible for chromosomal crosslinking and chromosomal anchoring by binding to the telomeres via the shelterin protein TRF2 (telomere repeat binding factor 2) [31,32]. cHL is, however, characterized by genomic instability [8,33,34], and it has been previously demonstrated that 3D direct telomere-TRF2 interaction is severely disrupted in H- and RS-cells [35]. In order to explore whether the telomeres of H and RS cells would bind to the internal lamin A/C structure, we performed immuno-staining for lamin A/C combined with telomere Q-FISH. Our results revealed that lamin is binding the telomeres in a low percentage in both cell types. Future studies on the direct interaction between lamin A/C and TRF2 will be necessary to understand if lamin A/C is actively involved in disruption of the telomere-TRF2 interaction process.

In summary, our results show that lamin A/C spatial organization may be instrumental in the transition from mononuclear H- to bi- and multi-nucleated RS-cells and that the technology of 3D analysis holds the potential for becoming an invaluable predictive tool in the clinical management of this complex disease. However, live cell imaging for visualization of lamin will be necessary to follow not only the formation of the internal lamin A/C structures but to confirm their impact in the multinucleation process.

## 4. Materials and Methods 

### 4.1. Lymphocyte Isolation and Stimulation

Peripheral blood lymphocytes (PBLs) were isolated from peripheral blood from healthy donors through Ficoll-gradient centrifugation (Ficoll-Paque^TM^ Plus, 17-1440-02, GE Healthcare, Little Chalfont, UK). Blood was diluted with PBS (3.5:1) and settled with a ratio of 1.5:1 on Ficoll. The obtained buffy coat was collected and washed twice in a PBS solution. Stimulation of B-lymphocytes was obtained culturing PBLs with 50μM of Lipopolysaccharides (LPS) (SIGMA, L2630, St. Louis, MO, USA) for 72 h. Isolated resting and LPS-activated PBLs were then placed onto poly-l-lysine (SIGMA, p8920, St. Louis, MO, USA) coated slides.

### 4.2. Cell Lines

Three different EBV-negative cHL derived cell lines were used for this study: Nodular Sclerosis cHL derived cell lines HDLM-2 and L-428, and Mixed Cellularity cHL derived cell line L-1236 (DSMZ, Braunschweig, Germany). The HDLM-2 and L-428 cell lines were grown in RPMI-1640 medium, supplemented with 20% fetal bovine serum (FBS), 1% l-glutamine, 1% sodium pyruvate, and 1% penicillin–streptomycin (reagents from Invitrogen/Gibco, Burlington, ON, Canada). The L-1236 cell line was grown in RPMI-1640 medium, supplemented with 10% FBS, 1% l-glutamine, 1% sodium pyruvate, and 1% penicillin–streptomycin. Cells were incubated at 37 °C with 5% CO_2_ in a humidified atmosphere. Fresh slides were prepared before every experiment spreading the cells onto poly-l-lysine coated slides.

### 4.3. Immunohistochemistry

Primary Anti-Lamin A (rabbit polyclonal, ab26300, Abcam Ltd., Cambridge, UK) and secondary Goat Anti-rabbit Alexa 488 (Molecular Probes, Waltham, MA, USA) antibodies were used for immunohistochemical analysis of lamin A/C at a dilution of 1:200 and 1:500 respectively in 4%BSA/4× SSC blocking solution. Primary Anti-Lamin B1 (rabbit polyclonal, ab16048, Abcam Ltd. UK) and secondary Goat Anti-rabbit Cy3 (AP187C, Sigma Chemical, St. Louis, MO, USA) antibodies were used for immunohistochemical analysis of lamin B1 at a dilution of 1:200 and 1:500 respectively in 4%BSA/4× SSC blocking solution. The cells were fixed in 3.7% formaldehyde/1× PBS and permeabilized with 0.1% Triton-X 100. 1× PBS washes were performed to wash the solutions off. Slides were then incubated with primary antibody for 45 min at 37 °C, humidified atmosphere. 1× PBS washes were performed to wash away the extra primary antibody. Incubation with secondary antibody was performed for 30 min at 37 °C, humidified atmosphere. 1× PBS washes were performed to wash away the extra antibody. DNA of the nuclei was counterstained with DAPI. Vectashield was used as mounting medium to prevent photo-bleaching of the sample.

### 4.4. Lamin A/C Patterns Quantitative Analysis

The quantitative measurements of the lamin A/C patterns have been performed on the cHL derived cell line HDLM-2, on 30 H and 30 RS cells from three independent experiments. 2D images of H and RS cells representing the identified pattern were selected and used for the quantitative analysis on ZEN Blue Version 2.3 Software (Carl Zeiss, Jena, Germany). The Draw Spline Contour tool of ZEN was used to manually select two distinct areas: (i) the external lamin A/C fluorescent signal (I_e_) and (ii) the internal lamin A/C fluorescent signal (I_i_). Total external lamin A/C signal intensity and total internal lamin A/C signal intensity were calculated. A ratio of the resulting intensities (I_e_/I_i_) was calculated for every cell. The obtained ratios from cells having the same pattern were averaged and compared with the ratios obtained for the other patterns.

### 4.5. cHL Patient Samples

We analyzed 12 primary Hodgkin’s lymphoma paraffin embedded pre-treatment lymph node samples from patients diagnosed with cHL. The patients’ information are summarized in Table 3. The patients were consented to participate into this research, which was approved by the research ethics board at the Jewish General Hospital in Montreal (protocol 16-016).

Formalin-fixed, paraffin-embedded tissue slides (5 μm thickness) were deparaffinized at room temperature in xylene until the paraffin was completely dissolved away, and then placed in 100% ethanol. The slides were subsequently rehydrated in a descending gradient of ethanol-water solutions to 50% ethanol, and transferred to PBS before fixation in 3.7% formaldehyde/1× PBS. The immuno-staining protocol for lamin A/C applied was the same one used for the Hodgkin derived cell lines, with the addition of an antigen-retrieval treatment before the permeabilization in Triton-X. For this step, we incubated the slides in the Target Retrieval Solution (DAKO, S1700, Santa Clara, CA, USA) for 20 min at 90 °C, and then 20 min at room temperature (RT).

### 4.6. Immuno-Staining for Lamin A/C/Telo-Q-FISH

The combined quantitative 3D lamin A/C-telomere immuno Q-FISH protocol used in this study has been previously described by Knecht and Mai in 2017 [36]. In addition to the immuno-staining procedure, Q-FISH was performed using Cy3- labeled peptide nucleic acid (PNA) probe (Dako, Glostrup, Denmark). Slides were placed in 70% formamide/10 mM, then washed in 0.1× SSC at 55 °C. An additional wash in 2× SSC/0.05% Tween 20 was performed. A second incubation with primary and secondary antibodies was performed before DAPI counterstaining.

### 4.7. siRNA Silencing and Western Blot

Lamin A/C expression was monitored by Western Blot after siRNA lamin A/C transfection in different concentration (12.5 nM, 25 nM and 50 nM) for 24 h (Figure 5A), 48 h (Figure 5B), 72 h (Figure 5C) and 96 h (Figure 5D). siRNA lamin A/C transfection with 50 nM was also performed on two additional time points—120 h and 144 h (Figure 6)—to investigate the complete kinetics of siRNA-mediated lamin A/C silencing. siRNA Scramble was used as a negative control and Cyclophilin B was used as a loading control. The fold-decrease for the siRNA is relative to the negative control (Scramble for each concentration) and the Scramble is relative to control without transfection (Control). All numbers are indicated under each column. These experiments were performed in triplicate using cells from different passages.

### 4.8. 3D Image Acquisition

3D conventional imaging of 30 H, 30 RS, and 30 Lymphocytes was performed using ZEISS Axio Imager.Z2 (Carl Zeiss, Toronto, ON, Canada) with a cooled AxioCam HR B&W, FITC, Cy3 and DAPI filters in combination with a Planapo 63x/1.4 oil objective lens (Carl Zeiss). 60 z-stacks were imaged for every fluorophore of every cell with a 200 nm step among the stacks. Images were obtained using AxioVision 4.8 (Carl Zeiss), deconvolved using the constrained iterative restoration algorithm with Theoretical PSF and Clip Normalization, and rendered using the Maximum Module.

The automated image acquisition of interphase nuclei was performed using the ScanView system [Applied Spectral Imaging (ASI)], using a Zeiss Imager Z2 microscope with a Basler CCD camera. For scanning purposes, the microscope was equipped with a motorized nine-slide stage (Märzhäuser, Wetzlar, Germany). The 3D-images were acquired with dry 40× objective and a 0.6× c-mount (Olympus, Tokyo, Japan) taking 9 focal planes per cell. The axial sampling distance between planes, Dz, was 300 nm. Exposure times were constant at 1 ms (DAPI) and 100 ms (FITC) throughout the experiments. The tissue sample mode with cell circularity setting of 1.4 was used to enable segmentation of touching cells and cell detection. Cells touching the border of the field of view were excluded. Approximately 1000 were scanned and analyzed. For data acquisition and analysis the software modules GenASIs and SpotScan were used.

3D SIM imaging was performed using Zeiss PS.1 Elyra microscopy system equipped with 63x 1.4 NA objective lens and IXon 885 EMCCD Camera (Andor, Oxford, UK). Images were acquired using 1.518 refractive index (RI) immersion oil. 405 nm laser was used for excitation of the DAPI channel. SIM grating period of 28 µm was used. The step between z-planes (Δz), was set at 0.091 µm. The images were reconstructed with ZEN 2012 black edition software (Carl Zeiss, Jena, Germany) with Noise filter set to −3.0 and deactivation of the Baseline Cut option. Pixel size resulted 40 nm in the reconstructed image.

### 4.9. Image Analysis

For co-localization of lamin A/C and telomeres, following deconvolution of the images, a minimum of 30 H and 30 RS cells from three independent experiments on the HDLM-2 cell line were processed using NIH Image J Software (version 1.52e) and Tools for Analysis of Nuclear Genome Organization (TANGO) software (version 0.97) [25,26]. All structures were segmented using the stock segmentation; background was subtracted for Cy3 signals with subtract background 2D. Signal quantification was performed for FITC (lamin A/C) and Cy3 (telomere) signals and simple geometric measurements were taken for the nuclei and Cy3 signals. Co-localization was determined through the overlap between FITC signal of lamin A/C and Cy3 signal of the telomeres.

For telomeres’ analysis of siRNA treated cells and controls, deconvolved images were converted into TIFF files and exported for 3D analysis using the TeloView software program (version 2.0, 3D Signatures Inc., Toronto, ON, Canada) [37]. TeloView has been used for the analysis of the following parameters: (i) total number of telomeres’ signals; (ii) total number of telomeres’ aggregates; (iii) a/c ratio, which represents the cell cycle-dependent nuclear telomere distribution, where values close to 1 indicate cells are in G0/G1 phase, while values bigger than 1 are in S/G2 state; (iv) nuclear volume; (v) average telomeres’ signal intensity; (vi) total telomeres’ signal intensity.

The granulometry analysis of the DNA structure, DNA-free space and all computations were performed using the DIPimage toolbox for Matlab (version R2012a, MathWorks, Natick, MA, USA) as described by Righolt et al. in 2014 [8].

### 4.10. Statistical Analysis

Chi-square (χ^2^) test was used to assess the differences among the frequencies observed for the different patterns in H and RS cells. The telomeric parameters (number of telomeres, number of telomere aggregates, nuclear volume, a/c ratio, average and total telomere signal intensity) were compared between siRNA treated and Scramble H and RS cells, and among siRNA treated H and RS cells from different time points using a nested factorial analysis of variance (ANOVA) or two way ANOVA. Multiple comparisons using the least-square means tests followed in which interaction effects between two factors were found to be significant. Two-sided, two-sample Kolmogorov–Smirnov test was used to determine statistical significance of the differences measured in the granulometry analysis. Significance levels were set at *p* = 0.05.

## 5. Conclusions

Lamin A/C spatial distribution in H and RS cells from cHL is characterized by an aberrant shape and the presence of internal lamin structures and points of protein accumulation. These internal structures subdivide the nuclei into different sub-compartments. Due to the involvement of lamin A/C in regulating mitosis, these new findings suggest a potential role of lamin A/C in the multinucleation process from H to RS cells.

## Figures and Tables

**Table 1:** Means and frequencies of the lamin A/C 3D pattern observed in cHL-derived cell line HDLM-2. 30 H and 30 RS cells from three independent experiments were analyzed using the deconvoluted 3D reconstructions and divided according to the lamin A/C 3D pattern shown.

Lamin A/C Pattern	Mean ± SD	Percentage
Hodgkin Cell Pattern 0	5 ± 3	15.48%
Hodgkin Cell Pattern A	10 ± 1	29.15%
Hodgkin Cell Pattern B	14.67 ± 4.51	42.16%
Hodgkin Cell Pattern C	4.33 ± 3.51	11.63%
Hodgkin Cell Pattern D	0.67 ± 1.15	1.59%
Bi-nuclear Reed-Sternberg	19.67 ± 8.74	57.66%
Tri-nuclear Reed-Sternberg	7.33 ± 0.58	22.27%
Tetra-nuclear Reed-Sternberg	3.33 ± 1.15	10.29%
Multi-nuclear Reed-Sternberg	3 ± 4.36	9.79%

**Table 2:** Quantitative analysis of lamin A/C patterns in H and RS cells. 30 H and 30 RS cells of the cHL-derived cell line HDLM-2 from three independent experiments were analyzed. Total external lamin A/C signal intensity (I_e_) was divided by the total internal lamin A/C signal intensity (I_i_) to obtain a ratio. Resulting ratios and S.D. are shown.

Lamin A/C Pattern	I_e_/I_i_	S.D.
Hodgkin Cell Pattern 0	1.88	1.17
Hodgkin Cell Pattern A	2.65	1.15
Hodgkin Cell Pattern B	3.65	2.12
Hodgkin Cell Pattern C	4.02	2.66
Hodgkin Cell Pattern D	5.43	0.04
Bi-nuclear Reed-Sternberg	2.96	1.07
Tri-nuclear Reed-Sternberg	3.57	1.26
Tetra-nuclear Reed-Sternberg	4.50	3.37
Multi-nuclear Reed-Sternberg	4.55	2.95

**Table 3:** Clinical data of patients diagnosed with cHL. Abbreviations: ABVD—doxorubicin, bleomycin, vinblastine, dacarbazine; CHLVPP—chlorambucil, vinblastine, procarbazine, prednisone; EBV—Epstein Barr Virus.

Case	Gender	Age at Diagnosis	Stage	Type of Chemotherapy	Relapse	EBV Status	Lamin A/C Fluorescent Signal
1	Male	24	IV	ABVD	N	−	−/−
2	Female	55	III	ABVD	Y	−	−/+
3	Female	25	I	ABVD	N	+	−/−
4	Male	55	III	ABVD	Y	−	+++
5	Male	47	I	ABVD	N	+	+++
6	Female	75	IV	ABVD	N	−	−/+
7	Male	22	II	ABVD	N	−	+++
8	Male	19	II A	ABVD	N	−	−/+
9	Male	50	III A	ABVD	N	−	+++
10	Male	37	I A	ABVD	N	+	+++
11	Male	85	III	CHLVPP	Y	−	−/−
12	Male	28	IA	ABVD	N	−	−/+

## Supplementary Materials

The following are available online at http://www.mdpi.com/2072-6694/10/9/286/s1, Figure S1: Example of lamin B1 protein staining in cells from Hodgkin’s lymphoma (HDLM-2), Figure S2: Lamin B1 immunostaining of lymphocytes from PB of a healthy donor, Figure S3: H and E, CD30 and lamin A/C staining in primary Hodgkin’s lymphoma paraffin embedded pre-treatment lymph node tissues from patients diagnosed with cHL, Figure S4: Lamin A/C expression in primary Germinal Centre (GC) centrocytes from paraffin embedded reactive tonsil tissue from healthy controls, Table S1: Means of total area and total lamin A/C signal intensity alongside the frequencies of the lamin A/C 3D patterns observed in cHL-derived cell line HDLM-2. 1000 cells were automatically scanned and manually divided according to the lamin A/C 3D pattern shown, Table S2: Means and frequencies of the lamin B1 3D pattern observed in cHL-derived cell line HDLM-2. 30 H and 30 RS cells from three independent experiments were analyzed using the deconvoluted 3D reconstructions and divided according to the lamin B1 3D pattern shown. Videos S1–S5: H Cell Pattern (0,A,B,C,D), Video S6: RS Cell Bi-nuclear, Video S7: RS Cell Multi-nuclear, Video S8: RS Cell Tetra-nuclear.
